# Atlanta Society of Medicine

**Published:** 1890-01

**Authors:** 


### ATLANTA SOCIETY OF MEDICINE.

A regular meeting on December 3d, '89, was called to order by President Cooper.

The following interesting cases were reported:

Dr. Elkin reported a case of perineal section. The patient (age 18) was seen with Dr. Todd, on November 23d, 1889. An examination revealed two strictures of large calibre in the pendulous portion, and one of small calibre in the membraneous portion of the urethra. Two months previous another physician had divided the meatus to the normal size of the urethra, and some effort had been made to dilate the strictures. The case was complicated with several false passages at the seat of the deep strictures, and with two perineal abscesses pointing on either side of the anus, near the tuberosities of the ischii. On account of the false passages and perineal swelling great difficulty was experienced in getting anything into the bladder A small, deep urethral syringe was finally introduced and a perineal section was made with this instrument as a guide. The strictures in the anterior portion of the canal were divided by Otis' dilating urethrotome. The abscesses were opened and the patient made a good recovery.

Dr. Gaston stated that on one occasion he found great difficulty in locating the urethra in external urethrotomy.

Dr. Cooper reported a case very similar to that of Dr. Elkin, except the abscesses were connected with the urethra.

Dr. Nicolson reported a case of gunshot injury in a boy aged 12, received while handling the gun by the barrels and striking his dog with the butt. The entire charge passed into the left thigh, carrying the contents of the pocket, such as rocks, collar button, etc., into the wound. The opening of entrance was about two inches in circumference, and circular. The femoral artery lay pulsating in the inner border of the wound uninjured, and the lower part of the neck of the femur was carried away, though the bone was not fractured. The anterior crural nerve and its branches were destroyed for two inches of their course. Wound was cleansed and dressed with moist antiseptic dressings. The charge did not pass through the limb, but was buried in the muscles.

---

AT the annual meeting of the Atlanta Society of Medicine, held on December 17th, '89 the following gentlemen were elected officers for the ensuing year:

President—Dr. Nicolson.
Vice-President—Dr. McRae.
Secretary—Dr. Huzza.
Treasurer—Dr. Van Goitdsnoven.
Corresponding Secretary—Dr. Harris.
Reporters—Drs. Stockton, Kennedy and Joye.

The treasurer's report showed the Society to be in a very flourishing condition, with a handsome surplus in the treasury.

